# DNA Enrichment Methods for Microbial Symbionts in Marine Bivalves

**DOI:** 10.3390/microorganisms10020393

**Published:** 2022-02-08

**Authors:** Qiqi Li, Yu Chen, Si Zhang, Yuanjiao Lyu, Yiyang Zou, Jie Li

**Affiliations:** 1 CAS Key Laboratory of Tropical Marine Bio-Resources and Ecology, South China Sea Institute of Oceanology, Chinese Academy of Sciences, Guangzhou 510301, China; liqiqi19@mails.ucas.ac.cn (Q.L.); zhsimd@scsio.ac.cn (S.Z.); yjlv_dr_em@outlook.com (Y.L.); zouyiyang18@mails.ucas.ac.cn (Y.Z.); 2 Southern Marine Science and Engineering Guangdong Laboratory (Guangzhou), Guangzhou 511458, China; chenyu@gmlab.ac.cn; 3 University of Chinese Academy of Sciences, Beijing 100049, China; 4 Innovation Academy of South China Sea Ecology and Environmental Engineering, Chinese Academy of Sciences, Guangzhou 510301, China

**Keywords:** bivalves, microbial DNA enrichment, microbial abundance, microbial composition

## Abstract

High-throughput sequencing is a powerful tool used for bivalve symbiosis research, but the largest barrier is the contamination of host DNA. In this work, we assessed the host DNA reduction efficiency, microbial community structure, and microbial diversity of four different sample pre-treatment and DNA extraction methods employed in bivalve gill tissue samples. Metagenomic sequencing showed the average proportions of reads belonging to microorganisms retrieved using PowerSoil DNA extraction kit, pre-treatment with differential centrifugation, pre-treatment with filtration, and HostZERO Microbial DNA kit samples were 2.3 ± 0.6%, 2.5 ± 0.2%, 4.7 ± 1.6%, and 42.6 ± 6.8%, respectively. The microbial DNA was effectively enriched with HostZERO Microbial DNA kit. The microbial communities revealed by amplicon sequencing of the 16S rRNA gene showed the taxonomic biases by using four different pre-treatment and DNA extraction methods. The species diversities of DNA samples extracted with the PowerSoil DNA extraction kit were similar, while lower than DNA samples extracted with HostZERO Microbial DNA kit. The results of this study emphasized the bias of these common methods in bivalve symbionts research and will be helpful to choose a fit-for-purpose microbial enrichment strategy in future research on bivalves or other microbe–invertebrate symbioses.

## 1. Introduction

Bivalves are widely distributed from freshwater to marine environments and from coastal areas to the deep sea [1,2]. Many members of Bivalvia such as Mytilidae, Vesicomyidae, Solemyidae, Thyasiridae, and Lucinidae have been shown to coexist with microorganisms [3,4,5]. Symbioses between bacteria and marine bivalves were discovered 40 years ago at hydrothermal vents on the Galapagos Rift [6] and were later discovered in cold seeps [7], whale falls [8], sunken wood [9], and shallow-water coastal sediments [10]. The symbioses between bivalves and microorganisms allow both hosts and microbes to colonize and thrive in otherwise inhospitable habitats [11,12]. Symbiotic bacteria play a crucial role in mussel cell homeostasis [13,14], immune response [15,16], and metabolite and substrate exchange [17]. In return, the host provides microbes with metabolite, shelter, and population control [18,19].

Due to the important economic value and ecological benefits of bivalves, it is essential to identify the microorganisms in bivalves and uncover their roles. Given many symbiotic microorganisms that cannot be cultivated in vitro, the current research on bivalve symbionts mainly relies on culture-independent technologies including nano-scale secondary ion mass spectrometry (NanoSIMS) [20], high-throughput sequencing [17], matrix-assisted laser desorption/ionization MSI (MALDI-MSI) [21], and synchrotron-radiation based microcomputed tomography (SRμCT) [22]. The widely used high-throughput sequencing technology allows us to investigate the diversity of microorganisms and the mechanisms of symbiosis in mussels without the need for prior cultivation [17,23]. Though high-throughput sequencing is powerful for symbiosis research, minimizing the contamination of host DNA is still a challenge [24]. To deal with this problem, either removing the host DNA or deeper sequencing is required to capture microbial signals swamped by host DNA [25]. However, high-throughput sequencing technologies have platform-specific inherent biases and shortfalls. The accuracy and coverage for GC-rich regions and long homopolymer stretches are still problematic [26]. In addition, the short reads produced by most current platforms limit the accuracy of the data [27]. The ever-growing number of sequences presents a challenge for computational methods that compare DNA sequences to the database of microbial genomes and filter eukaryotic host sequences [28,29]. Therefore, it is necessary to reduce the complexity of the sample and separate the host and symbiotic microorganisms as much as possible.

At present, obtaining bivalve symbiotic microbial DNA includes two major approaches: one is extraction of genomic DNA from bivalve samples directly using CTAB method [17,30] or commercial kits, such as PowerSoil DNA extraction kit, which was commonly used in previous studies [31,32]. These methods might result in the quantity of host DNA deeply exceeding microbial DNA, limiting the sensitivity of nucleic acid-based microbial diagnostic systems [33,34,35]. Another approache is that using differential centrifugation [36,37] and filtration [23,38] pre-treatment methods or host DNA depletion kit [23] to enrich microbial cells in consideration of the contamination of host DNA. Differential centrifugation and filtration are easy-handling methods that can be achieved at the sampling site. The combination of differential pelleting and rate-zonal density gradient centrifugation has been successfully used for the enrichment and separation of symbionts from mussels and subsequent proteomic analyses, for which the number of identified symbiont proteins in enriched samples increased by 9.3% compared to those from unenriched tissues [18]. The combination of filtration and DNase I digestion was used to reduce host DNA effectively in clam sample, and no contamination of host nuclear DNA was detected in DNA sample [39]. 

In recent years, several commercial host DNA depletion kits have been available. The NEBNext Microbiome DNA Enrichment kit can selectively bind and remove CpG-methylated host DNA, keeping microbial DNA with low CpG methylation. According to the differences between eukaryotic hosts and microbial cells, several commercial kits, such as QIAamp DNA Microbiome and HostZERO Microbial DNA, enrich microbial DNA through differential lysis of host and microbial cells and removal of the released DNA of the host prior to total microbial DNA purification. While the NEBNext Microbiome DNA Enrichment kit is not fit for invertebrate samples due to the low levels of invertebrate genome methylation [40]. Employing QIAamp DNA Microbiome kit acquired higher bacterial DNA yields from oyster tissue, while the microbial diversity was reduced [23]. Though the HostZERO Microbial DNA kit could effectively deplete host DNA in clinical samples [41], food [42], and coral [43], it has not been applied in host DNA depletion for bivalve samples. In addition, Ahannach et al., 2021 found that different microbial DNA enrichment methods can introduce some biases into the microbial diversity profile and cause potential taxonomic composition differences in skin and saliva samples [41]. To date, the efficiency of these different methods for depleting host DNA from bivalve tissues and the bias introduced by these methods to the composition of these samples have rarely been evaluated [23].

In order to identify preferred methods to enrich microbial DNA from bivalve samples and evaluate the bias introduced by different methods, we chose *Perna canaliculus* gill tissue as material because it is easy to acquire from markets and the result obtained from it is representative. We collected and enriched bacteria and archaea cells from *P. canaliculus* gill tissues using differential centrifugation and filtration and then extracted DNA from the enrichment. The commercial kits, i.e., HostZERO Microbial DNA kit and PowerSoil DNA extraction kit, were used to extract DNA from gill tissues. The host DNA depletion efficiency was evaluated, and the bias of these methods was tested by comparing the retrieved microbial communities.

## 2. Materials and Methods

### 2.1. Sample Collection and Treatment

The *P. canaliculus* were collected from a commercial farm in Guangzhou, China (*n* = 15). The bivalves were transported to the laboratory immediately. Shell length, width, and depth were recorded for each mussel. The shell was cleaned with sterile imidazole-buffered saline (IBS) and wiped with 75% alcohol to reduce contamination of gill tissues by surface microorganisms. The gills were carefully transferred to a sterile centrifuge tube. Sterile IBS was added into the tube, and then the tube was gently shaken on a vortex mixer for ten seconds. After that, liquid was discarded followed by five rinses of gill tissue in sterile IBS. Gill tissue (~20 g) was homogenized in IBS by using 0.5 and 1.5 mm beads in TissueRuptor (Gering, Tianjin, China). The homogenized samples were divided into twelve samples each containing approximately 1.7 g of homogenized tissue sample and used for DNA extraction.

### 2.2. DNA Extraction

A total of four methods were used in this study as follows (Table 1): 

Method 1 (PS): DNA was extracted from homogenized tissue directly using a PowerSoil DNA extraction kit (Qiagen, Hilden, Germany) according to the manufacturer’s instructions.

Method 2 (PC): Differential centrifugation was carried out according to the previous method [37]. The homogenate was slowly centrifuged in a swing-out rotor (5 min, 500× *g*, 4 °C) to pellet host nuclei and gill tissue debris and then the supernatant were transferred into a new 15 mL tube and centrifuged for 10 min at 15,000× *g* and 4 °C to pellet all remaining cells. The final pellet was washed with IBS and kept on ice. DNA was extracted from the pellet collected through differential centrifugation using a PowerSoil DNA extraction kit according to the manufacturer’s directions.

Method 3 (PF): Homogenized tissue sequentially through a 40 µm cell strainer (Biolgix, Shawnee Mission, KS, USA) and a 5 µm polycarbonate membrane (Millipore, Billerica, MA, USA)). The flowthrough (prokaryotic cell enriched) was collected and centrifuged at 4 °C, 15,000× *g* for 5 min. The final pellet was washed with IBS and kept on ice. The PowerSoil DNA extraction kit (Qiagen, Hilden, Germany) was used according to the manufacturer’s instructions.

Method 4 (HZ): Host cells were removed from homogenized tissue using the Host Depletion Solution in HostZERO microbial DNA kit (Zymo Research, Orange, CA, USA). Microbial cells were lysed in ZR BashingBead™ Lysis tubes (0.1 and 0.5 mm) containing ZymoBIOMICS Lysis Solution. The manufacturer’s instructions were followed.

In addition, DNA was extracted from nuclease-free water (Qiagen, Hilden, Germany) by each DNA extraction method (*n* = 3) and served as negative control to determine possible reagent and laboratory contamination [44]. All samples were performed in triplicate. The yield and quality of extracted DNA were measured using a NanoDrop ND-1000 spectrophotometer (Thermo Fisher Scientific, Waltham, MA, USA) and agarose gel electrophoresis.

### 2.3. Metagenomic Sequencing and Data Analysis

To determine the proportion of microbial DNA compared to host DNA, metagenome sequencing was performed on the MGI-SEQ 2000 platform (BGI, Shenzhen, China), generating 2 × 100 bp paired-end reads. High-quality clean reads were obtained by removing reads with adaptor sequences, unknown nucleotides more than 0.5%, or low-quality bases more than 20% with SOAPnuke v 2.1.0 [45]. The clean reads were used for BLASTn searches and annotation against the NCBI non-redundant nucleotide sequence (Nt) database with an E-value cut-off of 10^–5^ (parameters: -task dc-megablast -evalue 1 × 10^−5^ -num_threads 10 -max_target_seqs 1). The reads annotated microorganisms divided by the overall annotated reads was the proportion of retrieved microbes. The obtained metagenomic datasets have been deposited in the NCBI Sequence Read Archive under Bioproject accession number PRJNA793305.

### 2.4. 16S rRNA Gene Amplicon Sequencing and Analysis

The bacterial and archaeal community composition in each extract was identified by high throughput sequencing of the hypervariable V3–V4 region of the 16S rRNA gene. The barcoded primer sets 338F (5′-ACTCCTACGGGAGGCAGCA-3′) [46] and 806R (5′-GGACTACHVGGGTWTCTAAT-3′) [47] were used to amplify the bacterial V3-V4 region of the 16S rRNA genes. The hypervariable V3-V4 region of the archaeal 16S rRNA genes was amplified using barcoded primers 349F (5′-GYGCASCAGKCGMGAAW-3′) [48] and 806R (5′-GGACTACVSGGGTATCTAAT-3′) [49]. The PCR cycling conditions were as follows: 98 °C for 3 min, followed by 28 cycles at 98 °C for 30 s, annealing at 55 °C for 30 s, elongation at 72 °C for 40 s, and a final elongation at 72 °C for 5 min. PCR amplicons were purified with Agencourt AMPure Beads (Beckman Coulter, Indianapolis, IN, USA) and quantified using the PicoGreen dsDNA Assay kit (Invitrogen, Carlsbad, CA, USA). Subsequently, amplicon libraries were prepared and then pooled in equal amounts and 2 × 250 bp pair-end sequencing was performed using the lllumina NovaSeq platform (Illumina, San Diego, CA, USA). The 16S amplicon sequencing of bacteria and archaea in this study were deposited in NCBI Sequence Read Archive database under BioProject PRJNA793291 and PRJNA793303.

Raw sequences from experimental samples were imported into QIIME2-2020.2 to filter out high quality reads [50]. Paired-end Illumina reads were quality-filtered, denoised, and grouped into ASVs (amplicon sequence variant) using the DADA2 [51]. Taxonomy was assigned using the SILVA v132 database. For diversity estimates and comparisons between different samples, the sequences were randomly subsampled into the minimum number of sequences obtained from the sample with the poorest sequencing effort. To visualize microbiota composition, stacked barplots were constructed in ggplot2 (v.3.3.2) [52]. Alpha diversity indices were calculated using vegan package v2.5–6 in R [53]. Principal coordinates analysis (PCoA) was performed using vegan packages, based on the Bray–Curtis distance, to test and visualize the patterns in microbial community compositions. Statistical significance was evaluated according to the pairwise Bray–Curtis dissimilarity value between each sample with every other sample.

## 3. Results

### 3.1. Quality and Quantity of the Extracted DNA

The yield and purity of the extracted DNA were measured using Nanodrop (Table S1). Most of the extracted DNA had a 260/280 ratio of around 1.8, which were acceptable values. However, the DNA extracted directly with the HostZERO microbial DNA kit (method 4, HZ) showed lower 260/280 and 260/230 ratios, considering that the protein and guanidine residues had not been completely removed. The DNA yield showed that there was no significant difference among PC (17.7 ± 2.0 μg, method 2, microbes enriched using differential centrifugation), PF (18.4 ± 4.4 μg, method 3, microbes enriched using filtration), and PS (14.7 ± 0.8 μg, method 1, DNA extracted directly with the PowerSoil DNA Isolation kit) methods, while DNA directly extracted with the HostZERO microbial DNA kit had a much lower yield (0.04 ± 0.01 μg) (*p <* 0.001).

### 3.2. Efficiency of Host DNA Depletion

**Figure 1:**
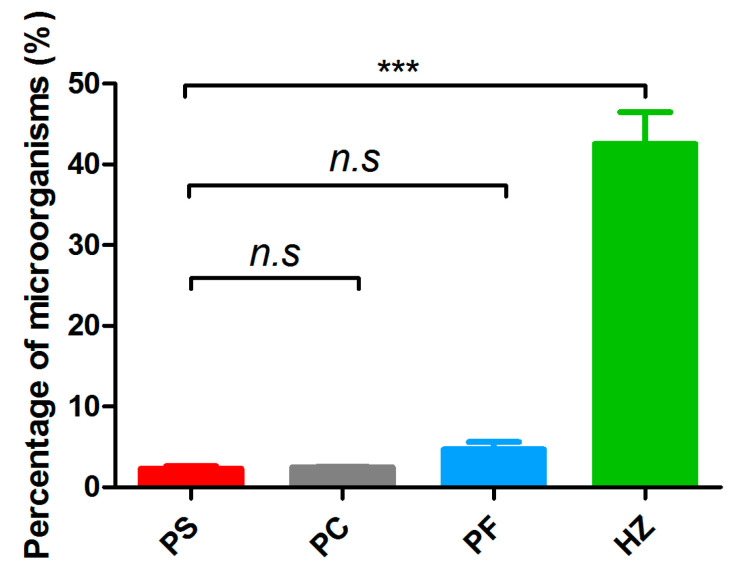
The proportion of sequences belonging to microorganisms in the metagenomic libraries. Significant differences (*p* < 0.001) across samples are indicated with “***”. “n.s”, not significant. PS, DNA was extracted directly with PowerSoil DNA extraction kit; PC, DNA was extracted with PowerSoil DNA extraction kit after differential centrifugation; PF, DNA was extracted with PowerSoil DNA extraction kit after filtration; HZ, DNA was extracted directly with HostZERO microbial DNA kit.

The proportion of microbial DNA component after host DNA depletion was evaluated. In total, 1 Gbp sequencing reads of each sample were obtained to evaluate the efficiency of host depletion. Annotation results showed that the proportions of sequencing reads affiliated with bacteria and archaea in the PS (2.3 ± 0.6%), PC (2.5 ± 0.2%), and PF (4.7 ± 1.6%) groups were similar (Figure 1). While the proportion of microorganisms in HZ group was up to 42.6 ± 6.8%, which was more than 9-fold increased in comparison to the other methods (*p <* 0.001) (Table S2).

### 3.3. Compositions and Diversities of Bacterial Communities Retrieved Using Different Methods

**Figure 2:**
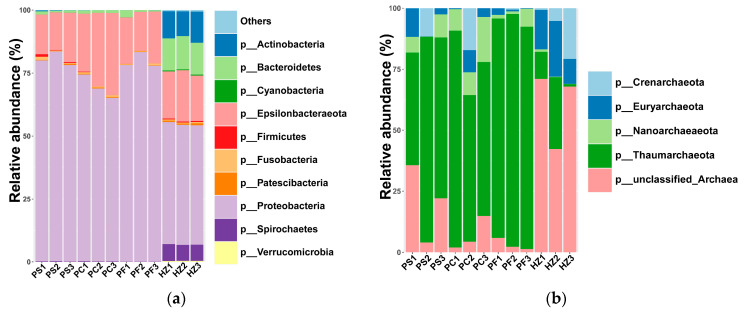
Stacked bar graphs showing the most common bacterial (**a**) and archaeal (**b**) phyla obtained using different microbial DNA enrichment methods. The top 10 bacterial phyla were shown in detail, and the rest are represented by “others”.

**Figure 3:**
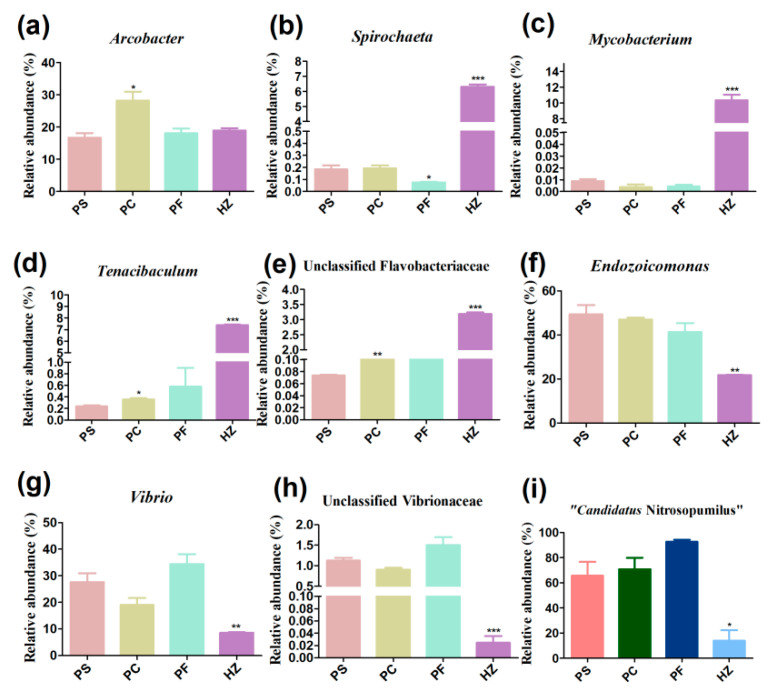
Relative abundances of the most abundant genera in each sample. Significant differences across PS and other samples are indicated with “*”. “*”, *p* < 0.05; “**”, *p* < 0.01; “***”, *p* < 0.001. (**a**) Relative abundance of *Arcobacter* in each sample. (**b**) Relative abundance of *Spirochaeta* in each sample. (**c**) Relative abundance of *Mycobacterium* in each sample. (**d**) Relative abundance of *Tenacibaculum* in each sample. (**e**) Relative abundance of unclassified Flavobacteriaceae in each sample. (**f**) Relative abundance of *Endozoicomonas* in each sample. (**g**) Relative abundance of *Vibrio* in each sample. (**h**) Relative abundance of unclassified Vibrionaceae in each sample. (**i**) Relative abundance of “*Candidatus* Nitrosopumilus” in each sample.

In addition to the assessment of host DNA depletion efficiency, we also compared the microbial compositions obtained using different methods. In total, 952,614 clean reads were obtained (Table S3). All of the obtained bacterial sequences could be clustered into 10,023 ASVs. In all bacterial communities, Proteobacteria, Epsilonbacteraeota, and Bacteroidetes constituted the most abundant phyla (Figure 2). Compared to the DNA directly extracted using the PowerSoil DNA Isolation kit (method1, PS), differential centrifugation (method 2, PC) significantly increased the relative abundance of *Arcobacter*, which belongs to Epsilonbacteraeota (*p <* 0.05) (Figure 3a). The pre-treatment method of filtration (method 3, PF) significantly decreased the relative abundance of *Spirochaeta* compared to DNA directly extracted using the PowerSoil DNA Isolation kit (Figure 3b). At the phylum level, the HostZERO microbial DNA kit (method 4, HZ) resulted in a significant decrease in the relative abundance of Proteobacteria and significant increase in the abundances of Bacteroidetes, Actinobacteria, and Spirochaetes (*p* < 0.05) in comparison with the PowerSoil DNA Isolation kit. Additionally, the relative abundances of *Mycobacterium*, *Spirochaeta*, *Tenacibaculum*, and unclassified Flavobacteriaceae were 11.5%, 7.0%, 7.8%, and 3.3%, respectively, in bacterial communities retrieved using HostZERO microbial DNA kit, which were higher than using the other three methods with < 1.0% relative abundance (*p* < 0.01) (Figure 3b–e). While the relative abundances of *Endozoicomonas*, *Vibrio*, and unclassified Vibrionaceae were decreased in the bacterial communities obtained by using HostZERO microbial DNA kit (Figure 3f–h). 

**Figure 4:**
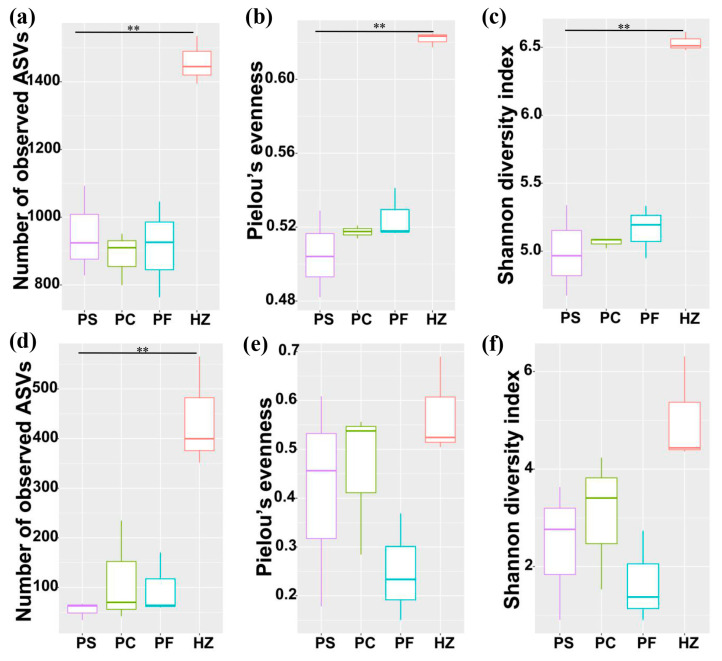
Alpha diversity estimates of the bacterial (**a**–**c**) and archaeal (**d**–**f**) communities. (**a**,**d**) ASV richness estimates (number of observed ASVs). (**b**,**e**) Pielou’s evenness estimates. (**c**,**f**) Shannon diversity indices. Significant differences (*p* < 0.01) across samples are indicated with “**”.

**Figure 5:**
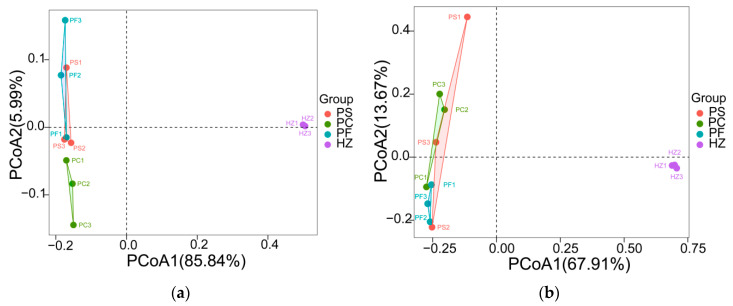
Comparison of bacterial (**a**) and archaeal (**b**) communities retrieved using different methods. The PCoA plot is based on a Bray–Curtis distance matrix of the 16S rRNA gene amplicon libraries.

The Alpha diversities of the bacterial communities retrieved using the PowerSoil DNA extraction kit, pre-treatment with differential centrifugation, and pre-treatment with filtration were similar, while they were obviously higher (*p* < 0.01) in the communities obtained by using the HostZERO microbial DNA kit (Figure 4a–c, Table S4). The comparison of the bacterial communities obtained using these four methods further showed that the communities obtained using the HostZERO microbial DNA kit formed a separate cluster and bacterial communities retrieved using PowerSoil DNA extraction kit, pre-treatment with differential centrifugation, and pre-treatment with filtration were similar (Figure 5a). Pairwise comparison of the communities retrieved using PowerSoil DNA extraction kit, pre-treatment with differential centrifugation, and pre-treatment with filtration showed 0.14–0.26 dissimilarities (Figure S1a). While in comparison to the communities retrieved using HostZERO microbial DNA kit, the dissimilarities were 0.67–0.69 (Figure S1a).

### 3.4. Compositions and Diversities of Archaeal Communities Retrieved Using Different Methods 

A total of 1,306,557 archaeal sequences (average 108,879 per sample) were identified in the twelve samples (Table S5) and clustered into 1848 ASVs. The major archaeal phyla were Thaumarchaeota, Euryarchaeota, Nanoarchaeaeota, Crenarchaeota, and unclassified archaea in all samples (Figure 2b). The HostZERO microbial DNA kit significantly decreased the relative abundance of Thaumarchaeota (14%) compared with the other three methods (65% of PS, 71% of PC, and 92% of PF, *p* < 0.05 for t-tested); and at the genus level, the relative abundance of “*Candidatus* Nitrosopumilus”, a member of Thaumarchaeota significantly decreased (Figure 3i). Compared to directly using the PowerSoil DNA Isolation kit, homogenates pre-treated with differential centrifugation and filtration did not affect the archaeal community composition. Multiple pairwise comparisons among communities directly using the PowerSoil DNA Isolation kit, pre-treated with differential centrifugation, and pre-treated with filtration did not reveal any significant differences in archaeal diversity, richness, and evenness (*p* > 0.05). While archaeal richness was obviously higher (*p* < 0.05) in the communities obtained by using the HostZERO microbial DNA kit compared with communities obtained with the PowerSoil DNA Isolation kit (Figure 4d, Table S6). As shown in Figure 5b, samples formed two major clusters and archaeal communities retrieved using PowerSoil DNA extraction kit, pre-treatment with differential centrifugation, and pre-treatment with filtration were similar. Pairwise comparison of the communities retrieved using PowerSoil DNA extraction kit, pre-treatment with differential centrifugation, and pre-treatment with filtration showed 0.35–0.56 dissimilarities (Figure S1b). While in comparison to the communities retrieved using HostZERO microbial DNA kit, the dissimilarities were 0.99 (Figure S1b).

## 4. Discussion

The combined application of next-generation sequencing technologies and multi-omics approaches will further enhance our understanding of symbiosis in the bivalve. However, the presence of an overwhelming amount of host DNA is one of the most important problems to be addressed. The collection and enrichment of bacteria and archaea from bivalve gills could be performed according to the distinct sizes of the host and microbial cells [23]. Bivalve gill epithelial cells are on average 20 μm wide, whereas a typical bacterial cell is ~2 μm [22]. We accordingly filtrated tissue homogenate across a 40 μm cell strainer and filters with pore sizes of 5 μm [54] to enrich bacteria and archaea cells. The results showed that the pre-treatment method of filtration did not significantly increase the proportion of microbial-derived reads in the metagenomic libraries. This result was similar with the enrichment effect on oyster and saliva samples [23,55]. It was likely due to the high amount of host DNA released during the homogenization process, which filtrated through the membrane filter and was collected with the microbial cells together.

Differences in size or density allowed the separation of bivalve and microbial cells using differential centrifugation [37]. In this study, the differential centrifugation was employed to separate microbial cells from host cells, while this pre-treatment did not reduce the amount of host DNA. Pathirana et al. [23] also found that differential centrifugation did not increase the bacterial DNA yield in oyster samples. It was likely that the host and microbes were not completely separated during the homogenization process, and the microbes precipitated in the lower layer along with the host cells and were discarded during differential centrifugation. These results may suggest that only using differential centrifugation cannot achieve gill tissue microbial enrichment; higher separation resolution is required.

Although the methods PS, PC, and PF achieved much higher DNA yields in comparison to the method HZ (i.e., extraction of DNA using HostZERO Microbial DNA kit), the first three methods did not significantly increase the proportion of microbial-derived reads in the metagenomic libraries. However, by using the HostZERO Microbial DNA kit, reads mapped to microbial sequences were increased nine folds, which is essential for shotgun metagenomic sequencing analysis. These results suggest that the high DNA yields acquired using methods PS, PC, and PF are probably attributed to the involvement of host (gill tissue) DNA. The HostZERO Microbial DNA kit has also been reported to have had good host depletion efficiency in coral and clinical samples [24,41,43]. That means selective lysis of eukaryotic host cells followed by removal of exposed DNA could be an effective method of reducing host-aligned sequencing reads, and it is also suitable for enriching microbial DNA from bivalve gill tissues.

Compared with the PowerSoil DNA extraction kit, the HostZERO Microbial DNA kit could significantly reduce the proportion of host DNA as well as increase the diversity of both bacteria and archaea detected. This result was consistent with previous studies on respiratory tract samples [56]. As the proportion of host DNA was reduced, some low-abundance microorganisms could be detected and the microbial community showed higher species diversity [57]. Increased species diversity is beneficial for understanding the bivalve symbiotic microbiome.

In this study, we further compared the microbial community compositions revealed by using these four methods. The results showed that though the species diversities among communities retrieved using filtration and differential centrifugation pre-treatment and PowerSoil DNA extraction kit were similar, the filtration and differential centrifugation pre-treatments introduced slight taxonomic bias in revealing microbial composition. The filtration pre-treatment significantly reduced the relative abundance of *Spirochaeta* in comparison to the DNA directly extracted using the PowerSoil DNA Isolation kit. The cell length of most *Spirochaeta* spp. was 9–30 μm [58], which exceeds the pore size of the 5 μm membrane filter, thus this group might be removed during filtration. 

The HostZERO Microbial DNA kit could significantly increase the relative abundances of *Mycobacterium* and *Tenacibaculum* in comparison to DNA extracted using the PowerSoil DNA Isolation kit. *Mycobacterium* and *Tenacibaculum* are currently believed to be related to mortality events in bivalves [59,60]. These results suggest that the HostZERO Microbial DNA kit could improve the analytical sensitivity of pathogen detection. Moreover, we found that the DNA extracted with HostZERO Microbial DNA kit had lower relative abundances of most gram-negative taxa in comparison to PowerSoil DNA extraction kit. This bias has also been observed in the saliva microbial community [41]. It might be that the relative abundance of gram-negative bacteria was “diluted” by the DNA of gram-positive bacteria. In addition, as the cell wall of gram-negative bacteria is thin, some might be lysed together with host cells and the released genomic DNA were removed. Therefore, the limitations of this method must be considered when specifically focusing on gram-negative groups. In addition, the HostZERO Microbial DNA kit could significantly decrease the relative abundances of the major group, Thaumarchaeota, in comparison to DNA extracted using the PowerSoil DNA Isolation kit, while abundant unclassified archaea were detected in the libraries constructed from DNA extracted using the HostZERO Microbial DNA kit. These results imply that diverse unrecognized archaea are rare groups in *P. canaliculus* gill tissues, which could only be detected when the host DNA was removed from the whole genomic DNA as much as possible.

The results in this study showed that different microbial DNA enrichment methods caused microbial taxonomic bias in depicting bivalve-associated microbial compositions. At present, obtaining bivalve symbiotic microbial DNA includes CTAB method [17], commercial kits [31], differential centrifugation [36,37] and filtration [23,38] pre-treatment methods, or host DNA depletion kit [23]. The many variations in DNA extraction methodology decrease consistency and comparability between studies on bivalve microbiome and may confuse the results. Therefore, it is essential to apply the consistent protocol throughout the study of bivalve to reduce potential batch effect associated with the experimental procedure of microbiome analysis. In addition, the bias should be considered in microbiome analysis if data is from different experimental sources.

## 5. Conclusions

In conclusion, this article brings attention to the importance of choosing an appropriate sampling method and nucleic acid extraction method for studies of the bivalve microbiome. Results of this study suggest that the HostZERO Microbial DNA kit is a desirable method for enriching microbial DNA from Bivalvia gill tissue samples. The pre-treatment methods of filtration and differential centrifugation did not significantly enrich bivalve microbiome. The uncovered Alpha diversities of bacterial and archaeal communities in gill tissue of bivalve were largely affected by DNA extraction method. DNA samples extracted using the HostZERO Microbial DNA kit had higher bacterial diversity than PowerSoil DNA extraction kit, while the HostZERO Microbial DNA kit showed a bias against gram-negative taxa. The results of this study suggest that it is critical to realize that microbial DNA extraction methods introduce a taxonomic bias that cannot entirely be avoided.

## Figures and Tables

**Table 1:** The workflows of four methods. The homogenized gill tissue of *Perna canaliculus* was divided into four groups and the total DNA was extracted using four different methods, including direct extraction using PowerSoil DNA extraction kit (PS), extraction using PowerSoil DNA extraction kit after differential centrifugation (PC), extraction using PowerSoil DNA extraction kit after filtration (PF), and direct extraction using HostZERO microbial DNA kit (HZ). IBS represents imidazole-buffered saline.

Sample	Method	Differential Enrichment Steps	DNA Extraction Kit
*Perna canaliculus* gill tissue homogenate	PS	No enrichment	DNeasy Power Soil Kit
	PC	1. Centrifugation a. 5 min at 500× *g* b. 10 min at 15,000× *g* 2. Final pellet washing with IBS	DNeasy Power Soil Kit
	PF	1. Filtration a. 40 μm cell strainer b. 5 μm polycarbonate membrane 2. Centrifugation 5 min at 15,000× *g* 3. The final pellet was washed with IBS	DNeasy Power Soil Kit
	HZ	No enrichment	HostZero Microbial DNA Kit

## Data Availability

Data can be obtained upon request to the authors.

## Supplementary Materials

The following supporting information can be downloaded at: https://www.mdpi.com/article/10.3390/microorganisms10020393/s1, Figure S1: Pairwise Bray–Curtis dissimilarities of bacterial (a) and archaeal (b) communities retrieved using different methods; Table S1: DNA yield and purity; Table S2: Numbers of reads belonging to prokaryotic microorganisms and eukaryote host in the metagenomic libraries; Table S3: Summary on raw data processing of bacteria; Table S4: Alpha diversity metrics of bacterial communities revealed using four DNA extraction methods; Table S5: Summary on raw data processing of archaea; Table S6: Summary on raw data processing of archaea.
