# Supplementary material for: DNA Enrichment Methods for Microbial Symbionts in Marine Bivalves
Source: Microorganisms. 2022 Feb 8;10(2):393. doi: 10.3390/microorganisms10020393 (PMC8878965; doi:10.3390/microorganisms10020393)
Supplement: Supplementary file 1 [file microorganisms-10-00393-s001.zip › microorganisms-1572678-supplementary.pdf]

Supplementary Material

1. Supplementary Figures:

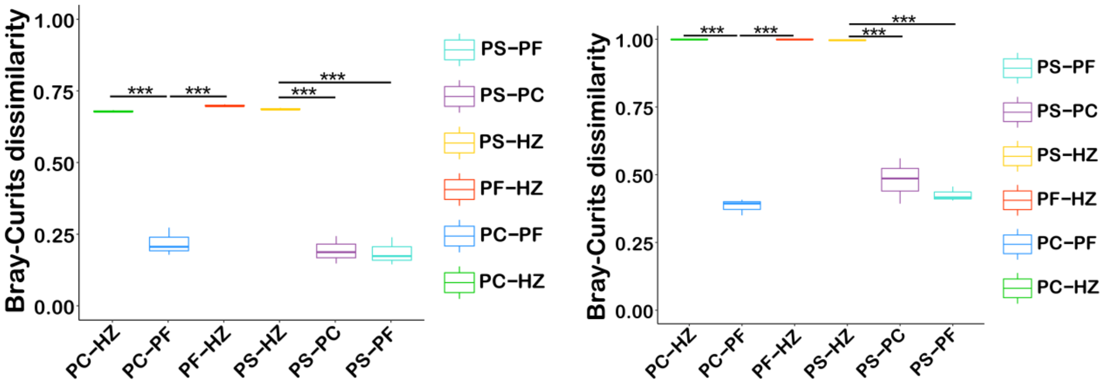

(**a**) (**b**)

Figure S1. Pairwise Bray-Curtis dissimilarities of bacterial (a) and archaeal (b) communities retrieved using different methods. Significant differences ($P < 0.001$) across samples are indicated with "***". PS, DNA was extracted directly with PowerSoil DNA extraction kit; PC, DNA was extracted with PowerSoil DNA extraction kit after differential centrifugation; PF, DNA was extracted with PowerSoil DNA extraction kit after filtration; HZ, DNA was extracted directly with HostZERO microbial DNA kit.

2. Supplementary Tables:

Table S1. DNA yield and purity. PS, DNA was extracted directly with PowerSoil DNA extraction kit; PC, DNA was extracted with PowerSoil DNA extraction kit after differential centrifugation; PF, DNA was extracted with PowerSoil DNA extraction kit after filtration; HZ, DNA was extracted directly with HostZERO microbial DNA kit.

Sample	DNA quantity (μg)	260/280	260/230
PS1	13.84	1.81	2.05
PS2	15.44	1.82	2.13
PS3	14.72	1.81	2.11
PC1	19.36	1.85	2.46
PC2	15.44	1.86	2.43
PC3	18.24	1.86	2.49
PF1	23.28	1.86	2.48
PF2	17.20	1.86	2.48
PF3	14.80	1.86	2.48
HZ1	0.05	1.33	0.64
HZ2	0.04	1.05	0.53
HZ3	0.04	1.32	0.33

Table S2. Numbers of reads belonging to prokaryotic microorganisms and eukaryote host in the metagenomic libraries. PS, DNA was extracted directly with PowerSoil DNA extraction kit; PC, DNA was extracted with PowerSoil DNA extraction kit after differential centrifugation; PF, DNA was extracted with PowerSoil DNA extraction kit after filtration; HZ, DNA was extracted directly with HostZERO microbial DNA kit.

Samples	PS1	PS2	PS3	PC1	PC2	PC3	PF1	PF2	PF3	HZ1	HZ2	HZ3
Sum prokaryotic microorganism Reads	5043	6160	7009	6148	7017	6921	7819	19262	20026	223281	286637	562299
Sum Eukaryote Reads	166541	315780	337128	265372	257910	273417	260303	302520	370617	388471	406783	565407

Table S3. Summary on raw data processing of bacteria. PS, DNA was extracted directly with PowerSoil DNA extraction kit; PC, DNA was extracted with PowerSoil DNA extraction kit after differential centrifugation; PF, DNA was extracted with PowerSoil DNA extraction kit after filtration; HZ, DNA was extracted directly with HostZERO microbial DNA kit.

SampleID	Input	Filtered	Denoised	Merged	Non-chimeric
PS1	112942	101538	100034	97562	75701
PS2	120240	108655	107673	105919	84686
PS3	118603	106393	105275	103551	81928
PC1	108151	97295	95990	93163	70266
PC2	97840	88644	87699	86100	65591
PC3	108319	97826	96722	94774	66857
PF1	110756	99311	98101	95817	73438
PF2	110695	99597	98879	97657	73364
PF3	120600	108376	106932	104899	69246
HZ1	141646	128344	126079	121363	100248
HZ2	134030	122036	120022	116046	98656
HZ3	137833	125035	122637	117455	92633

Table S4. Alpha diversity metrics of bacterial communities revealed using four DNA extraction methods. PS, DNA was extracted directly with PowerSoil DNA extraction kit; PC, DNA was extracted with PowerSoil DNA extraction kit after differential centrifugation; PF, DNA was extracted with PowerSoil DNA extraction kit after filtration; HZ, DNA was extracted directly with HostZERO microbial DNA kit.

Sample	Chao1	Simpson	Shannon	Pielou	Observed _species	Goods _coverage
PS1	1172.23	0.929153	5.33866	0.528919	1092.6	0.996897
PS2	959.007	0.897039	4.67322	0.482085	828.2	0.996883
PS3	1028.95	0.914268	4.96612	0.504071	924.2	0.99681
PC1	1012.14	0.919808	5.0842	0.513888	951.2	0.997423
PC2	844.992	0.922628	5.02185	0.520828	799	0.998041
PC3	946.445	0.918447	5.08816	0.51763	910	0.998076
PF1	1118.92	0.922909	5.19456	0.517869	1046	0.997236
PF2	850.238	0.920244	4.9491	0.516748	764	0.997624
PF3	976.587	0.936877	5.33261	0.541115	926	0.997679
HZ1	1651.39	0.966698	6.61309	0.624829	1534.8	0.995877
HZ2	1498.17	0.965774	6.511	0.623337	1394.4	0.996455
HZ3	1537.17	0.963198	6.47943	0.61728	1444.9	0.996363

Table S5. Summary on raw data processing of archaea. PS, DNA was extracted directly with PowerSoil DNA extraction kit; PC, DNA was extracted with PowerSoil DNA extraction kit after differential centrifugation; PF, DNA was extracted with PowerSoil DNA extraction kit after filtration; HZ, DNA was extracted directly with HostZERO microbial DNA kit.

SampleID	Input	Filtered	Denoised	Merged	Non-chimeric
PS1	136665	122263	121730	120796	119327
PS2	120258	105611	105323	104993	104831
PS3	139087	123656	123540	122834	120117
PC1	132185	116996	116964	116812	113675
PC2	117477	105430	104674	103036	88185
PC3	131716	118692	118134	117255	110546
PF1	128603	115177	114925	114284	110381
PF2	135707	118680	118327	118142	116976
PF3	133601	116047	115903	115705	112034
HZ1	133623	121316	120704	112860	106750
HZ2	141319	128702	127668	121321	103703
HZ3	130067	117588	117037	104075	100032

Table S6. Alpha diversity metrics of archaeal communities revealed using four different DNA extraction methods. PS, DNA was extracted directly with PowerSoil DNA extraction kit; PC, DNA was extracted with PowerSoil DNA extraction kit after differential centrifugation; PF, DNA was extracted with PowerSoil DNA extraction kit after filtration; HZ, DNA was extracted directly with HostZERO microbial DNA kit.

Sample	Chao1	Simpson	Shannon	Pielou	Observed _species	Goods _coverage
PS1	63.5283	0.869436	3.63209	0.608591	62.6	0.999967
PS2	34	0.275308	0.908369	0.178551	34	1
PS3	67.0233	0.672651	2.76463	0.456242	66.7	0.99998
PC1	42	0.380607	1.53396	0.284656	41.9	0.99999
PC2	235.264	0.796647	4.23467	0.537674	234.9	0.999922
PC3	69.95	0.777626	3.40736	0.556293	69.8	0.999983
PF1	172.878	0.562225	2.73407	0.368749	170.6	0.999888
PF2	64.1	0.177691	0.900644	0.150165	63.9	0.999994
PF3	59.635	0.315633	1.37619	0.23346	59.5	0.999984
HZ1	405.031	0.800524	4.36045	0.504521	399.7	0.999743
HZ2	570.209	0.966215	6.30563	0.689716	565.1	0.99968
HZ3	352.944	0.871201	4.43494	0.524337	351.7	0.999873
